# Manipulations of List Type in the DRM Paradigm: A Review of How Structural and Conceptual Similarity Affect False Memory

**DOI:** 10.3389/fpsyg.2021.668550

**Published:** 2021-05-31

**Authors:** Jennifer H. Coane, Dawn M. McBride, Mark J. Huff, Kai Chang, Elizabeth M. Marsh, Kendal A. Smith

**Affiliations:** ^1^Colby College, Waterville, ME, United States; ^2^Department of Psychology, Illinois State University, Normal, IL, United States; ^3^The University of Southern Mississippi, Hattiesburg, MS, United States

**Keywords:** false memory, DRM paradigm, activation monitoring theory, fuzzy trace theory, global matching

## Abstract

The use of list-learning paradigms to explore false memory has revealed several critical findings about the contributions of similarity and relatedness in memory phenomena more broadly. Characterizing the nature of “similarity and relatedness” can inform researchers about factors contributing to memory distortions and about the underlying associative and semantic networks that support veridical memory. Similarity can be defined in terms of semantic properties (e.g., shared conceptual and taxonomic features), lexical/associative properties (e.g., shared connections in associative networks), or structural properties (e.g., shared orthographic or phonological features). By manipulating the type of list and its relationship to a non-studied critical item, we review the effects of these types of similarity on veridical and false memory. All forms of similarity reviewed here result in reliable error rates and the effects on veridical memory are variable. The results across a variety of paradigms and tests provide partial support for a number of theoretical explanations of false memory phenomena, but none of the theories readily account for all results.

## Introduction

Over the past 25 years, the Deese-Roediger-McDermott (DRM; Deese, 1959; Roediger and McDermott, 1995) paradigm for studying experimentally induced false memories has been used in thousands of studies. To give a simple example, as of January 2021, a search on SCOPUS indicated the 1995 paper has been cited over 2,450 times. The basic findings of this corpus of research suggest that studying lists of related words (e.g., *butter, knife, slice*) elicits reliable false recall and recognition of a non-presented critical item (CI, e.g., *bread*).

The false memories obtained using this paradigm are robust across testing formats [see Gallo (2006, 2010), for reviews], emerge after encoding as few as four or five related list items (Coane et al., 2007, in preparation), and persist weeks to months following study [Seamon et al., 2002; Coane et al., manuscript in preparation; but see Colbert and McBride (2007)]. The *DRM false memory illusion* is highly replicable, both between and within participants (Zwaan et al., 2018), indicating that awareness of the paradigm does not eliminate the effect. Indeed, administering a warning to avoid recalling the CI prior to study (Gallo et al., 1997; Huff et al., 2012) or promoting more distinctive encoding processes [e.g., Israel and Schacter, 1997, see Huff et al. (2015), for a review and meta-analysis] reduces, but does not eliminate, the illusion. When queried, participants are highly confident in their accuracy for these falsely remembered items (e.g., Roediger and McDermott, 1995) and will make confident source attributions to the non-presented lures (Payne et al., 1996). Further underscoring the strength of these false memories, when assessing the phenomenological reports given by participants, they are likely to identify the CI as *remembered* rather *than known*, suggesting that specific episodic details are associated with the retrieval of the lure (Brainerd et al., 2003; Geraci and McCabe, 2006). Furthermore, the effect is present across age groups, languages, and in individuals with dementia and other forms of neurological damage (Balota et al., 1999). In fact, normative studies have generated DRM lists across several languages, including Spanish (Anastasi et al., 2005; Beato and Díez, 2011), French (Brédart, 2000), Italian (Senese et al., 2010), Portuguese (Albuquerque, 2005), and Romanian (Horoitǎ and Opre, 2020). DRM studies have also been conducted in Chinese (Guo et al., 2004) and Japanese (Kawasaki and Yama, 2006) languages, replicating the false memory effect in non-alphabetical languages. Despite obvious language differences across these studies, patterns found in the DRM paradigm are remarkably consistent: Manipulations that affect false memory rates using English materials show similar patterns in other languages. In sum, this work highlights the malleability of memory and the importance of examining how related words can give rise to high-confidence memory errors.

## Defining Similarity

The use of list-learning paradigms that are dependent upon similarity between list items and CIs to explore false memory has underscored several critical findings about the contributions of similarity and relatedness in memory phenomena more broadly. Such issues are at the core both of research in episodic memory and in understanding the organization of knowledge in semantic memory. In fact, these questions have been examined, in one way or another, for decades, if not since the beginning of traditional memory research. In his seminal study, Ebbinghaus (1885/1913) purposely selected meaningless syllables to avoid the potential contamination of meaning-based information in recall. The use of meaningless stimuli, which, by definition, are unrelated to one another, was a hallmark of early memory research (McGeoch, 1942) as scholars attempted to uncover memory principles and processes.

However, meaning-level information, broadly defined, exerts a powerful effect on many cognitive processes. Cognitive systems are highly adept at applying meaning to information in the environment through pattern recognition processes and the application of top-down processes (i.e., prior knowledge, context information). Organizational processes such as the Gestalt principles underscore how readily the cognitive system uses surface level features, such as proximity and similarity, to create a coherent representation of the environment. Such processes give rise to phenomena such as visual illusions and pareidolia (i.e., detecting faces in non-face stimuli; Ichikawa et al., 2011), and are critical for how the mind organizes a complex environment. These organizational principles further extend to memory systems, where reliance on structures such as schemas and categories support encoding and retrieval processes by simplifying the amount of information to which an individual must attend. For example, categorization allows one to quickly retrieve previously known information about a novel member of a category and reduces the need for re-learning (Bruner et al., 1956). Relying on schemas and scripts similarly minimizes the amount of effort and attention necessary for navigating the world. Such reliance on prior knowledge systems and structures, however, does come at a cost: namely, the introduction of errors through a reconstructive memory process, which occurs when previous experiences are retrieved (Bartlett, 1932; Bergman and Roediger, 1999; Schacter, 2001; Roediger and DeSoto, 2015). Reconstructive processes might be more likely to occur when information is poorly encoded, due perhaps to inattention, or forgotten, due to decay or interference, leading to increased reliance on existing knowledge structures to “fill in the gaps” in a memory. This is illustrated in many types of memory errors, from schema-driven errors (e.g., falsely recalling books in an office; Brewer and Treyens, 1981) to misinformation effects (Loftus, 2005). The DRM paradigm is similarly dependent on these established knowledge systems stored in semantic memory, such that studying the list items results in the increased accessibility or familiarity of the CI due to the shared meaning between items.

Several other paradigms in memory research have underscored the powerful effects of relatedness in short- and long-term memory. For example, in short-term memory tasks, phonological similarity effects (Conrad, 1963; Baddeley, 1964; Wickelgren, 1965) refer to high error rates for phonologically and/or orthographically similar items and reflect the high reliance on surface-level features in short-term memory. In Baddeley's working memory model (Baddeley and Hitch, 1974), this is mediated via the active maintenance of verbal information in the phonological loop. When short sets of items from the same category are studied, the degree of proactive interference observed is dependent on the degree of similarity between sets (Wickens, 1970); this suggests that, in addition to phonological information, semantic information is also processed and preserved in short-term memory. Classic work examining long-term recall and recognition also highlights the powerful effects of relatedness on retention. For example, related word lists are recalled better than unrelated ones (Huff et al., 2011). Lists of categorically related words are not only recalled better than unrelated ones but show clustering effects such that the shared meaning provides organizational structure at retrieval (Bousfield, 1953). Such effects occur spontaneously or when category cues are provided at retrieval (Tulving and Pearlstone, 1966).

Clearly, as this brief (and selective) review highlights, similarity along multiple dimensions exerts powerful effects on memory. This raises the question of how similarity is defined. Characterizing the nature of “similarity and relatedness” can inform researchers about factors contributing to memory distortions and about the underlying networks that support episodic memory. An examination of the semantic memory literature reveals that operationalizing relatedness in terms of meaning is far from straightforward. One of the fundamental debates in the literature concerns whether relatedness is driven by lexical-level associations or by semantic similarity [see Kumar (2021), for a recent review]. The former refers to the types of associations in the language that are due to co-occurrence or other types of experience. For example, *cat* and *dog* are related because they tend to be encountered in similar contexts (e.g., both are house pets, or are found in idiomatic expressions such as “it's raining cats and dogs”). Items like *dog* and *leash* are related because of a functional association [although some researchers argue that functional relations are a form of semantic relatedness; see Lucas (2000), Wu and Barsalou (2009)]. Conversely, semantic similarity is defined as similarity in terms of overlap of primitive features or category membership. In this case, *cat* and *dog* are related because they generally share physical features (e.g., fur, claws, four legs) and belong to the same category. In an extensive review of the semantic priming literature, Hutchison (2003) concluded that automatic semantic priming, which refers to the facilitation observed when a target item (e.g., *dog*) is processed faster and/or more accurately when it is preceded by a related prime (e.g., *cat*) than an unrelated prime (e.g., *pen*), can occur following both associative relations and feature overlap. Overall, semantic priming tasks suggest that access to a target can be facilitated by associations and semantic similarity.

One of the most compelling lines of evidence in support of associative priming comes from mediated priming tasks. In such experiments, prime-target pairs are developed such that the prime and target are not directly related to each other, but indirectly related to a non-presented mediator that connects them (e.g., *lion*-*stripes*, in which the non-presented mediator is *tiger*; Balota and Lorch, 1986). Because mediated pairs do not share any features directly, they provide strong support for associative accounts of priming (Hutchison, 2003). Conversely, priming from synonyms and antonyms is consistent with feature overlap accounts of priming. The traditional DRM lists used in most research contain a mixture of semantic and associative relations and as such, are consistent with both semantic and associative accounts of priming. As we discuss below (see Theories of False Memory in the DRM Paradigm), whether false memories in the DRM paradigm are due to semantic and/or associative processes is at the center of theoretical debates about the mechanisms that give rise to errors. Careful manipulation of the type of relation between list items and CIs can refine these theories.

Although meaning-based similarity is a powerful determinant of memory errors, cognitive systems are also highly tuned to detecting and identifying patterns and similarity in terms of surface features. In addition to semantic priming, priming can also occur when primes and targets are related phonologically and/or orthographically (i.e., form priming). It is beyond the scope of this work to provide a full review of the literature on these forms of priming effects [for reviews see Rastle (2007), Farrell et al. (2012), Humphreys et al. (2016)]; such effects are robust in both spoken and written language. Whether priming is facilitatory or inhibitory depends on several factors, such as stimulus onset asynchrony, task demands, and masking, to name a few. However, there is consistent evidence that related primes do affect the time it takes to retrieve a target. One important element is that such effects are due to lexical level factors and are independent of meaning. Thus, form priming appears to be distinct from semantic priming.

Similarly, memory errors can emerge due to the encoding of formally or structurally similar items. For example, Koutstaal and Schacter (1997); Koutstaal et al. (1999, 2003) have repeatedly demonstrated false memories based on perceptual information for images (both meaningful and abstract), and Zeelenberg et al. (2005) reported false memories following the study of lists of non-words. Further, variants of the DRM paradigm using items that are related phonologically and/or orthographically have produced robust false memory rates (e.g., Sommers and Lewis, 1999; Watson et al., 2003). Thus, similarity between list items and CIs—in terms of meaning or surface features—result in memory errors. In the present work, we provide an overview and review of research using the DRM paradigm to examine several manipulations of “similarity.” The findings from this work can be used in testing theories for explaining false memory in this paradigm and further our understanding of how these factors affect memory more generally. We note that most of the work using verbal materials in this area has been conducted in English; where relevant, we include evidence from other linguistic and alphabetic systems.

## Theories of False Memory in the DRM Paradigm

There are several current theoretical explanations of false memory. The activation-monitoring framework (AMF; Roediger et al., 2001) emphasizes the role of automatic spreading activation in lexical/semantic networks (e.g., Collins and Loftus, 1975) that increases the accessibility or familiarity of the CI through shared pathways. In support of the role of activation-based processes, the degree to which list items and CIs are associated (backward associative strength, BAS) based on free association norms (Nelson et al., 2004), is the best predictor of false recall and the second-best predictor of false recognition (Roediger et al., 2001). In addition, evidence that false memories emerge under divided attention conditions (Peters et al., 2008), occur following incidental encoding tasks (Dodd and Macleod, 2004), and via the presentation of list item distractors during a recognition test (Coane and McBride, 2006), further supports the automatic nature of this process. The second process, source monitoring (Johnson et al., 1993), can result in the misattribution of this activation to a studied event rather than to the internal generation of the CI. Source monitoring is a controlled, resource-demanding process that is necessary to avoid errors and, when it fails, an increase in false memories is observed. Evidence in support of this process is found in studies that have shown a reduction in errors when an explicit warning to avoid critical intrusions is given (Gallo et al., 1997; McCabe and Smith, 2002; Neuschatz et al., 2003), for individuals with higher working memory (e.g., Watson et al., 2005), and younger (vs. older) adults (Balota et al., 1999), where the last two groups typically possess stronger memory monitoring capacities. Thus, the AMF includes two opponent processes: an error-increasing activation process and an error-reducing monitoring process.

Separately, fuzzy-trace theory (FTT; Brainerd and Reyna, 2002) presupposes that upon experiencing an event, two parallel traces are stored: a verbatim trace, which preserves item-specific and contextual information, and a persistent gist trace, which is based on the extraction of the general meaning of the encoded information. FTT attributes false memories to reliance on the gist trace based on the similarity between list items. CIs are consistent with the gist or thematic coherence of the list, leading to errors, whereas memory for list items can be supported by both gist and verbatim traces. Verbatim memory can serve to reduce errors through a process referred to as recollection rejection. Verbatim traces tend to decay more rapidly, whereas gist traces are persistent (e.g., Abadie and Camos, 2019). Thus, FTT incorporates an error-increasing mechanism (gist memory) and an error-reducing mechanism (verbatim memory/recollection rejection). Evidence consistent with FTT includes findings that false memories are more persistent than veridical memories [Toglia et al., 1999; Seamon et al., 2002; but see Colbert and McBride (2007)] and higher rates of false memories for lists with a stronger thematic coherence (Cann et al., 2011; Carneiro et al., 2014).

Finally, global matching models (GMM) such as MINERVA2 (Hintzman, 1986, 1988; Arndt and Hirshman, 1998) suggest that items are encoded in memory as feature vectors; related items share features, thereby leading to similar traces. The extent to which a retrieval probe matches vector traces stored in memory determines whether an item is recognized as studied or not. The feature matching process results in a familiarity echo signal that is stronger with more feature overlap between a probe and the memory traces. Feature overlap is summed for each study-test item comparison, which results in an activation value. Activation values are then summed across item comparisons to provide a familiarity level for each test probe. Because CIs, by definition, share features with all studied list items, when presented as probes they will likely elicit a strong level of familiarity due to the summed activation from the feature matches across the items, thereby leading to an incorrect old response. One advantage of these models is that they can readily account for false memories for non-words (Zeelenberg et al., 2005) or abstract images (Koutstaal et al., 2003), as they do not require pre-existing mental representations that would result in activation or gist extraction. Some also assume that when a test probe is compared with an encoded trace and overlapping features are found, all of the traces' features are activated as activation spreads from those features that overlap with the probe (Hintzman, 1986). Furthermore, these models have flexibility in defining what the features that are stored in memory traces are and include semantic features as well as surface level features.

A common factor across these theories is that they attribute an important role to similarity in veridical and false memory effects. As noted above, similarity can be defined in terms of lexical/associative properties (e.g., shared connections in associative networks), semantic properties (e.g., shared conceptual and taxonomic features), or structural properties (e.g., shared orthographic or phonological features). In the AMF, similarity is defined in terms of connections between nodes in lexical and semantic networks; in FTT, similarity is based on gist traces that are meaning-based; and in GMM, similarity emerges through shared features that are stored with each memory trace, broadly defined. Thus, exactly how similarity or relatedness is defined varies somewhat across theories. By manipulating the type of list and its relationship to a non-studied CI, we have explored the effects of varying types of similarity on veridical and false memory in a variety of memory tasks, assessing short- and long-term memory, using recall and recognition tasks, priming tasks, and in younger and older adults. Here, we review prior research manipulating list type. To preview our conclusions, all forms of similarity we have manipulated thus far have resulted in reliable error rates and the effects on veridical memory were variable, suggesting that multiple forms of relatedness support both accurate and erroneous memory.

## Decomposing Semantic and Associative Similarity

As noted above, a core question in the field of semantic memory concerns the nature of the representations, their organization, and, by extent, how relatedness is defined. If knowledge is primarily represented and organized along shared meaning, such as category relatedness, then shared primitive features (e.g., has skin, has four legs, breathes) would be critical in determining whether two concepts, and the words that represent them, are related. Conversely, if organization relies more on shared occurrence or broader principles of association, the connections between items would not depend on shared features as much as on more broadly defined relations and on co-occurrence in similar contexts (e.g., Landauer and Dumais, 1997). Manipulations of list type in the DRM paradigm have explored the question of what sorts of items elicit greater false memory. In broad terms, researchers have distinguished between categorical lists, in which the CI is either a member of the same category as the list items (e.g., a list of fruits with *apple* as the CI) or a category superordinate (e.g., a list of fruits with *fruit* as the CI), and associative lists, in which the list items and CIs are related based on free association norms (e.g., a list of items related to the CI *fruit* includes words such as *pie, basket*, and *bowl*). The first two types reflect semantic level relationships, whereas the latter relies more on associative and lexical relationships (although the two types of relationships are often confounded). In what follows, we use the term *categorical* to refer to relationships that depend on category co-occurrence or membership and shared features and the term *associative* to refer to relationships based on lexical co-occurrence.

Early research attempting to examine the roles of associative and categorical relations in the DRM found that associative lists resulted in higher false memory (Buchanan et al., 1999) and larger priming effects (Smith et al., 2002) than categorical lists. Other work (Dewhurst et al., 2007; Knott and Dewhurst, 2007; Knott et al., 2012) found that manipulations such as divided attention at encoding or manipulations of list presentation (e.g., blocked vs. random) exerted parallel effects on false memories for both list types. An important factor, however, is that BAS was higher in associative than categorical lists, introducing a potential confound. When BAS was matched, however, false memories were equivalent across list types, although the lists were not “pure” in that associative lists also included some category coordinates (Knott et al., 2012). In contrast, Park et al. (2005) reported higher rates of false recall and false recognition for associative than for categorical lists, even after controlling for BAS. Because BAS is a strong driver of false memory (Roediger et al., 2001) and some types of semantic relations, specifically synonyms, situation features, and taxonomic relations, are predictors of BAS (Cann et al., 2011), it can be difficult to tease apart the effects of association from those of shared features. To address this, we (Coane et al., 2016, 2020) developed novel lists that were matched in BAS but differed in whether they shared basic features. Non-categorically associated (NCA) lists consisted of associates to a CI that did not share features or come from the same category (e.g., *dog* CI with *bone, bark* list items), whereas categorical plus associative lists (C+A) lists had equivalent levels of BAS as the NCA lists, but also shared features and/or came from the same category (e.g., *dog* CI with *cat, wolf* list items). Thus, for each CI, we had two lists: one that shared features and one that did not. Norming studies confirmed that feature similarity was greater in the C+A lists than in the NCA lists. Importantly, the lists were matched not only in BAS, but along several other dimensions [e.g., word frequency, connectivity, semantic distance according to the Latent Semantic Analysis; Landauer and Dumais, 1997; see Coane et al. (2016), for details on list development and pre-testing]. We underscore the importance of matching the lists on key dimensions that are known to influence word recognition and lexical access. Although the relationship between access and activation is not fully understood, if a given item is accessed faster, this could result in more activation than an item that requires more time to access (cf. Westbury et al., 2002).

Under these conditions, C+A lists have reliably elicited higher false memory rates than NCA lists in both recall and recognition tasks [Coane et al., 2016, 2020; see also Montefinese et al. (2015)]. This suggests that, above and beyond activation as captured by BAS alone, semantic or feature similarity results in an increase in false memory, a phenomenon we refer to as a *feature boost*. One possibility is that shared features provide additional activation beyond that which comes from associations. Semantic priming research suggests that, when primes and targets are category coordinates, such as *goat*-*dog*, coordinates that are associated, such as *cat*-*dog*, generate larger priming effects than those that are not, a phenomenon referred to as an *associative boost* (Hutchison, 2003). In other words, associations and semantic similarity might exert additive effects on target or CI accessibility.

An alternative account of the feature boost is that there are differences in the extent to which error-reducing mechanisms, such as monitoring or recollection rejection, are effective. Although warnings generally reduce false memories, their effectiveness varies with the identifiability of the CI. Specifically, when the CI is easier to identify (Neuschatz et al., 2003; Carneiro et al., 2012; Huff et al., 2012, 2018) or when CIs are strongly thematically related to lists (Carneiro et al., 2014), warnings are more effective. To test whether the effect was due to differential CI identifiability, we (Coane et al., 2020) compared false memory for C + A and NCA lists after a warning or after participants were instructed to guess the CI. The guessing task provided an estimate of how accurately participants could identify the CI, as well as an implied warning given that participants were tasked with identifying a “missing” item. Although CIs from C + A lists were more difficult to identify, an explicit warning was equally effective at reducing errors in across list types. Importantly, when participants were able to correctly identify the CI, false alarms were equivalent across list types. Further, conditional analyses on recognition responses as a function of prior recall (one of the post-list task conditions) indicated that the feature boost only occurred when CIs had not been recalled previously. In other words, prior recall or correct guessing eliminated the feature boost effect, indicating that the feature boost in recognition only occurred when CIs were not explicitly identified either by guessing or prior recall. Thus, the feature boost does appear to be due, at least in part, to difficulties in discriminating, and thus rejecting, the CI.

In addition to our approach to separate the effects of associative and thematic similarity by holding BAS constant, other work has addressed the core question of how to tease apart semantic and associative similarity. Specifically, Brainerd et al. (2020) created a pool of 120 four-item DRM lists that varied widely in their mean BAS values and in their degree of semantic similarity between list items and CIs. All lists were normed to determine a measure of gist strength (GS) and empirically examine how BAS and GS jointly influence false recognition. They concluded that GS reliably predicted CI false recognition across levels of BAS, whereas mean BAS only predicted CI false recognition when mean GS was low.

Although in the present review we have focused on the comparison of categorical and associative lists, another manipulation is worth noting. Hutchison and Balota (2005) attempted to decouple associative strength from meaning or gist by developing lists in which the CI was a homograph (e.g., *fall*). In critical conditions, the list included items related to both meanings of the CI (e.g., *stumble, trip* as well as *autumn, leaves*). Thus, the meaning of the list items was associated to the CI at a lexical level, but the two halves of the list converged on two distinct meanings at the semantic level. False memories for these lists were compared to DRM lists in which all list items converged upon a CI with a single meaning. Critically, BAS was held constant between list types. False memories were equivalent across homograph lists and DRM lists both when homograph lists were blocked by meaning and when meanings were alternated within a list, indicating that divergent meanings exerted less of an effect than associations of any meaning, even when divergent meanings were less consistent with alternating presentations. More recently however, Huff et al. (2015) reported that blocked homograph lists could increase false recall relative to alternated lists, but only when the test was delayed. Taken together, these findings suggest that the influence of meaning and associative information on false memories is complex and variable.

Before discussing the theoretical explanations of these effects, we wanted to address some recent extensions of this work. First, we examined the effects of warnings and guessing the CI from NCA and C+A lists in a sample of older adults. Healthy aging is associated with preserved automatic processes and declines in controlled processing, and older adults have equivalent or elevated false memories compared to younger adults (e.g., Schacter et al., 1997; Balota et al., 1999; Liu and Cao, 2002; Huff and Aschenbrenner, 2018; Pansuwan et al., 2020). Older adults are also less likely than younger adults to benefit from a warning (McCabe and Smith, 2002), and there is evidence of different lifespan time courses for reliance on taxonomic (i.e., categorical) and associative or thematic information (Mirman et al., 2017; Belacchi and Artuso, 2018). Categorical information depends on more abstract and complex knowledge systems, and this organizational system emerges later in childhood compared to associative or thematic organization. As adults age, they benefit less from categorical information in recall compared to younger adults (Huff et al., 2011), whereas the benefits of associative or thematic information show less of a decline. Thus, it was possible that the feature boost would not be observed for older adults, who might rely, instead, more heavily on associative information.

In a study similar in design to Coane et al.'s (2020), a sample of 120 healthy older adults were assigned to one of the same four conditions in the study with young adults (Coane et al., in preparation): guess the CI, complete math problems (a no-retrieval control condition), complete a free recall task, or complete the free recall task with a warning. A final recognition test was then completed. The data were analyzed in conjunction with the younger adult data from Coane et al. (2020) to examine possible age differences. Older adults were less likely to correctly guess the CI for both C+A and NCA lists compared to younger adults and correct guesses were much higher for NCA lists than for C+A lists. Overall, younger and older adults' identification of the CIs was similarly affected by the different types of list relations. Free-recall performance revealed that warnings were effective for both younger and older adults in decreasing false recall of the CI, but again, there was no interaction with age or with condition. Veridical recall did not differ by age or condition, although, consistent with our earlier work, C + A list item recall was significantly greater than NCA item recall.

Older adults' performance on the final recognition test^1^ also mirrored the results in younger adults: Warnings reduced false recognition overall, significantly so relative to the no-warning recall condition, indicating they were still effective on a delayed test. Importantly, C + A lists elicited higher false alarms than NCA lists, and, although older adults had higher false alarms overall vs. younger adults, age did not interact with list type or condition.

Thus, older and younger adults do not differ in the extent to which the feature boost occurs. Furthermore, the lack of age differences suggests that the effect might be driven in large part by processes that are unaffected by age. CI identification, although lower in older than younger adults, appears to be one such process: If the CI is identified, participants appear to be able to use this information to reduce errors. However, because the CIs from C + A lists are less likely to be identified, the process is more likely to fail for these lists, resulting in higher error rates.

We have also examined the feature boost in short-term memory using a modified Sternberg task (Sternberg, 1966) with DRM lists (Xu et al., 2017). Participants studied lists of six items from a C + A or an NCA list and, after each list, responded to a single probe: the CI, a studied item from the set, or a non-studied item from the same list. False alarms to both CIs and non-studied items were higher for C+A than NCA lists; thus, a feature boost was found even under immediate test conditions when very short lists were studied. This suggests that the error-increasing effects of additional similarity in terms of features emerges rapidly and occurs when participants should be able to accurately monitor the source of an item's familiarity (because of the small set size and short delay). Overall, based on both our published and unpublished work, when CIs are both associatively related *and* share features with list items, false memories are greater than when there are no shared features present. The effect is robust across warning conditions, test type, and age.

Turning to how the three primary theoretical accounts can accommodate these findings, the AMF has difficulty explaining the difference in false memories across NCA and C + A lists given the equivalent BAS across list types. The automatic activation process, which is assumed to be predicted by BAS, should be equivalent. One possible explanation is that multiple sources of activation, lexical level associations and semantic level conceptual representations, are independent of one another and contribute separately to affect the activation or accessibility of the CI. This suggests that associations, as captured by free association norms, might be “missing” some important aspects of similarity or relatedness. In addition, the AMF cannot readily account for independent effects of forward associative strength (FAS, or the extent to which CIs elicit the list items in free association; Arndt, 2015), because the CI is not encoded, and thus, would not directly activate the list items, unless a complex process of mediated activation occurs. Conversely, this account can readily accommodate the findings from homograph lists, which appear to be driven by associative strength independent of meaning. As this review shows, similarity is a complex construct that reflects multiple layers and levels that might include several different features, including categorical, associative, lexical, orthographic, and semantic relations.

Conversely, FTT can quite readily accommodate the feature boost in long-term tests: Given that false memories are supported by gist traces, which depend heavily on thematic similarity, items sharing many features are likely to give rise to a stronger, more coherent gist. Thus, the benefit from shared features fits nicely with this theory. In addition, the evidence from Brainerd et al. (2020), that GS predicts false recognition whereas BAS only does so when GS is low, makes FTT a viable explanatory mechanism for these effects because GS is assumed to influence gist extraction and gist-sensitive retrieval processes. However, the fact that C + A CIs were harder to identify could be problematic, given that a stronger gist should be easier to identify. In addition, the feature boost effect in short lists with immediate tests is not consistent with this description because with short lists and delays, verbatim information should be more heavily relied on than gist for recognition responses, resulting in a reduction in the feature boost effect. Further, FTT has difficulty accommodating the results of the homograph lists: Theoretically, the mixed lists include two distinct gists, which should result in the storage of two weaker gist traces than lists with a single meaning convergence. However, the increased false recall for blocked over alternated lists on delayed but not immediate tests is consistent with FTT, suggesting that the stronger gist from blocked lists persists over time.

Unlike the other two models, GMMs can account for both sets of findings if the similarity between the stored and test traces is a function of both lexical level associations and primitive features. Because these models assume that many types of features (semantic associations, categorical features, and structural/lexicographic features) are stored during encoding and then compared with test probes during retrieval, any type of overlap would presumably increase activation, and in turn familiarity, for the CIs at test. For both the feature boost effect and false memories for homograph lists, these models describe retrieval responses based on activation of all the different types of features involved. Thus, GMMs predict a feature boost effect for C+A lists due to the larger amount of feature overlap with list item traces compared with NCA lists and a similar (or larger) amount of overlap in features for homograph lists compared with standard DRM lists.

## Manipulations of Associative Similarity: Mediated Lists

Despite its difficulty with the feature boost effect, the AMF fares better with results from studies examining mediated associations. One of the primary assumptions of the AMF is that false memories are the result of activation in lexical and semantic networks, which results in the indirect activation of the CI. Consistent with this, longer lists result in higher false alarm rates (Robinson and Roediger, 1997; Coane et al., 2007). Network models, such as Collins and Loftus's (1975), include dense networks of nodes, representing words and concepts, that are connected via pathways; the length of a pathway reflects the strength of the association between two nodes. Through a spreading activation mechanism, activation of one node results in an increase in activation of all nodes connected to it. Furthermore, this initial burst of activation continues to spread in a graded fashion throughout the network. Consistent with this assumption, mediated priming has been obtained for nodes separated by one (Balota and Lorch, 1986; Coane and Balota, 2011) or two (Chwilla and Kolk, 2002) mediators.

According to the AMF, then, studying a list of items that are directly related to an associate of the CI but are not themselves directly related to the CI, should result in increased activation of the CI. For example, for the CI *river*, directly related items include *water, boat*, and *swim*. The mediated list includes *faucet* (related to *water*, but not to *river* according to the Nelson et al., 2004, free association norms), *yacht* (related to *boat*, but not *river*), and pool (related to *swim*, but not to *river*). To test whether mediated lists can create false memories for such CIs, Huff and Hutchison (2011) had participants study mediated lists that were immediately followed by a free-recall test or arithmetic problems (i.e., a control condition). Following completion of several study list-recall/arithmetic cycles, all participants completed a final recognition test. On the initial test, false recall of mediated CIs was not found, however, reliable false recognition of mediated CIs was found on the final test. Additionally, mediated false recognition was greater for participants who completed initial recall tests vs. arithmetic problems. Thus, consistent with an implicit AMF, false memory for CIs was found in the absence of a list theme that is directly related to the CI and this pattern was restricted to the delayed recognition test. This pattern indicates that activation processes leading to mediated false memories are likely implicit in nature, do not occur on a recollection-heavy free-recall test, and only emerge on recognition when implicit familiarity-based processes contribute to memory responses. Mediated false recognition effects were also found across different study durations (3,000 ms vs. 500 ms) and following a guessing task where participants were asked to generate the mediated CI immediately after study. Correct guessing of mediated CIs was very low, providing additional evidence regarding implicit processes that occur with mediated lists.

In a second series of experiments, Huff et al. (2012), developed new mediated lists that utilized the same CIs in the original DRM lists [Roediger and McDermott, 1995; Stadler et al., 1999; see Huff et al. (2012), for details]. Thus, for each CI, we included a direct list (i.e., the traditional list of DRM associates) and a mediated list. Participants intentionally encoded each list and performed one of four tasks that were completed immediately after study: arithmetic problems, free recall, free recall with a warning about the nature of the DRM lists, or the guessing task. Again, very few participants were able to successfully guess the CI from the mediated lists, confirming the lack of a clear theme. Warnings were effective at reducing intrusions of the CI for direct lists, and false recall of mediated CIs was very low. However, on a final recognition task, CIs from mediated lists were indeed again recognized at significant rates, consistent with spreading activation processes in AMF. More importantly, warnings and the guessing task *increased* false alarms to mediated CIs relative to the recall task, whereas they decreased false alarms to direct CIs. This *ironic effect of guessing* suggested that the additional elaborative processing participants engaged in while trying to identify the CI increased its activation. However, the difficulty in identifying the CI rendered the warnings—both the explicit warning given in the recall condition and the implied warning in the guessing condition—ineffective. A highly similar pattern of results was observed in an aging sample (Coane et al., 2016). Because older adults typically show preservation of automatic processes along with declines in more controlled processes (Balota et al., 2000), the age invariance of the effect is consistent with the involvement of automatic activation processes.

More recently, we have further evaluated implicit activation processes of mediated lists using a semantic-priming paradigm. Specifically, we (Huff et al., 2021) presented participants with mediated study lists which were immediately followed by a test list in which the CI was presented in the first, third, or eighth test positions to assess the time course of CI activation following study. Participants were tasked with responding to test items using either a semantic-classification task (concrete vs. abstract decisions), a pronunciation task (reading test items aloud), or an old/new recognition task, in which the first two tasks assessed response latencies. Mediated false recognition patterns were again in evidence, and this pattern was consistent across test positions. Importantly, CI priming was also found across test positions and this pattern was greatest in the first test position, but declined (though remained statistically reliable) across the remaining test positions. Priming was similar on both classification and pronunciation tasks. Moreover, the shape of this priming pattern is consistent with spreading-activation processes which are argued to dissipate as time and the number of intervening items between prime(s) and target increases.

Overall, the results of our work examining mediated false memories is consistent with the AMF, given its reliance on spreading-activation mechanisms in existing semantic networks. The ironic effects of guessing and warnings provide further support for the role of monitoring processes: When monitoring fails due to a failure to identify the CI, false alarms increase. Although the activation process is assumed to be automatic and thus does not require attentional processes, sustained attention, and elaboration can increase or maintain activation (Neely, 1977). In addition, the sustained focus and retrieval attempt can strengthen an episodically formed network of associated items resulting in a more persistent trace when a retrieval mode is engaged (Tulving, 1983; Meade et al., 2007). Mediated false memory is challenging for FTT: The lack of thematic consistency renders the gist extraction process difficult if not impossible, as shown by the difficulty participants have in identifying the CI. Thus, it is not clear at present, how this theoretical approach can accommodate these findings. However, GGMs might be able to account for these results due to the activation of all of a trace's features when overlap with the test probe is found (Hintzman, 1986). If one assumes that mediated items share sufficient features with the CI itself to activate all the mediated items' features (e.g., the presentation of the CI *river* activates the “water” feature of list item *faucet*, which matches that feature of the CI), then the features that do overlap between them might be similar enough that the familiarity echo would be of sufficient strength to elicit an incorrect response. However, this assumption would lead one to predict lower rates of false alarms for mediated than direct list CIs but similar patterns for these rates across task conditions, and as the results of these studies show, there is a dissociable pattern of false alarms for mediated and direct list CIs across math, recall, and guessing/warning conditions (Huff and Hutchison, 2011; Huff et al., 2012). Thus, these results present some difficulty for the GMMs.

## Manipulations of Structural Similarity: Phonological and Orthographic Lists

Another form of similarity that results in significant false memories in recall and recognition is in terms of structural elements, namely shared phonemes and graphemes. Orthographically and/or phonologically similar items (hereafter, we refer to them as phonological associates for brevity) share spelling and/or pronunciation with the CI, generally in the absence of shared meaning. For example, the phonological list for the CI *sleep* includes items such as *sleet, keep*, and *steep*. The items often, but not necessarily, rhyme or share the first letters or letter clusters. Although the effects of phonological similarity on memory, particularly short-term memory, have been well-documented for decades, after the publication of Roediger and McDermott (1995) these effects were more systematically explored in long-term memory tasks, specifically false memory. Sommers and Lewis (1999) developed phonologically related lists using parameters from the Neighborhood Activation Model (NAM; Luce and Pisoni, 1998), which specifies that, in spoken word recognition, words are organized in similarity networks based on shared phonemes. In this model, a neighbor is an item that differs from another item based on a single phoneme; it is thus comparable to Coltheart et al.'s (1977) metric for quantifying orthographic neighbors (i.e., words that differ by a single grapheme).

Research using the DRM paradigm can inform researchers on the interactions between visual word recognition and memory processes, which are often examined separately (e.g., Westbury et al., 2002; Cortese et al., 2008; Hutchison et al., 2018). Specifically, this paradigm can be used to test predictions of word recognition models and results suggest some long-term maintenance or persistence of verbatim information. Many models of word recognition, whether spoken or written, assume that activation levels are determined by the relationship between words or word components (e.g., onset, syllable, coda). For example, Coltheart et al.'s (1993) dual process model assumes that similarity is defined within each lexicon; for example, in the orthographic lexicon, shared letters result in neighbor activation. In Plaut et al. (1996) model, which is based on parallel distributed processing, shared sublexical nodes become active and facilitate related target access.

Westbury et al. (2002) attempted to identify which specific sublexical components were most important in determining false memory rates by developing lists that shared the initial phoneme, the head (first two phonemes), or the rime (last two phonemes) with a monosyllabic CI. Head and rime related lists both elicited greater false recognition than initial phoneme lists, although all three list types elicited greater false alarms than completely unrelated controls. However, they did not find evidence suggesting the effect was driven by orthographic overlap, even though lists were presented visually. In contrast, Cortese et al. (2008) did find that false memories increased when lists had both high phonological and orthographic overlap, relative to when orthographic overlap was low. These effects have also been tested in Chinese (a logographic language where characters that differ in written forms can be pronounced similarly, as in English, but similarly written characters can also be pronounced differently). Qu and Ding (2010) found false recognition of CIs with phonologically associated Chinese lists. Furthermore, the degree of similarity did not appear to affect false memory because list items that shared the same syllables or items that only shared onsets or rhymes with the CI produced similar false alarm rates. Using orthographically associated Chinese lists, researchers also observed false recognition of CIs (Qu et al., 2010). In contrast to what was found with phonologically similar lists, false recognition rates were positively related to similarity, as reflected by higher false recognition rates when the CIs shared a larger orthographic overlap (i.e., tonetic symbol) with the list items. This differential pattern might originate from the logographic nature of Chinese language, such that the orthographic information is more associated with semantic content than the phonetic information (Lin and Han, 1999).

In further tests in English, Hutchison et al. (2018) found that adding a single item that shared the initial phonemes with a CI to eight associates in studied lists resulted in a significant boost in false recall and specifically in false *remember* responses to recalled CIs. This is consistent with models of spoken word recognition that suggest that initial phoneme information is important in narrowing a pool of potential neighbors during word recognition, such as the cohort model (Marslen-Wilson and Tyler, 1980). Of particular interest is the finding that the addition of the phoneme overlap item only affected false memory when it was presented after the associates and not before, consistent with the cohort model's predictions that context pre-activates potential targets and phonological information provides a selection mechanism.

In sum, lists consisting of neighbors of a CI have elicited robust false memories in both recall and recognition tasks, in some cases comparable to those obtained using semantically related lists. These findings have since been replicated many times (e.g., Oliver et al., 2016; Finley et al., 2017), confirming that structural similarity between list and lure items results in elevated false memory rates. In some cases, phonological lists yield similar false memory rates to semantic lists (e.g., Sommers and Lewis, 1999), but in other cases, phonological lists elicit lower rates of false memory (e.g., Watson et al., 2003). At very rapid presentation rates (i.e., 20 ms/item), phonological lists yield higher false recall rates than semantic lists; as presentation rates increase, the opposite occurs (McDermott and Watson, 2001; Ballardini et al., 2008). This suggests that early in processing, similarity in terms of surface features is more critical than similarity in terms of semantic properties. Given that long-term memory is heavily dependent on semantic coding and that surface level information is quickly lost (Sachs, 1967), this finding is not particularly surprising.

More recently, we (McBride et al., 2019) examined phonological false memories in a short-term memory task. The phonological coding of verbal material in short-term stores is one of its key characteristics; thus, we expected to find elevated errors to phonologically related CIs. Prior work (e.g., Coane et al., 2007; Atkins and Reuter-Lorenz, 2008; Flegal et al., 2010) had extended the DRM to short-term memory tasks, finding reliable error rates with lists as short as four or five items and test delays as short as 1 s. In our work, we directly compared semantic and phonological lists using a modified Sternberg (1966) task. Using materials from Watson et al. (2003), we created lists of six phonological and six semantic associates for each CI. After each list, a single probe was presented: the CI, a studied list item, or a non-studied associate from the same list. In addition to replicating findings of semantically driven false alarms that exceeded those for non-studied probes, false alarms to phonologically related CIs were much greater than those to semantically related CIs. Thus, in the short-term, memory errors in the DRM paradigm are more sensitive to structural/phonological similarity to the CI than to semantic similarity. Interestingly, when directly comparing short- and long-term memory for both list types, there is a reversal in false alarms to CIs: On a short-term test, phonological lists elicit more errors than semantic lists; but on a delayed test, semantic lists elicit more errors than phonological lists (Coane et al., in preparation).

There is also work examining the independent contributions of semantic and phonological similarity to CIs. Watson et al. (2003) combined lists of semantic and phonological items to create hybrid lists. These lists elicited a hyper-additive effect—false memories increased substantially when one, two, or three phonological associates were embedded in a list of semantic associates (list length was held constant across these conditions). Finley et al. (2017) further demonstrated that this hyper-additive effect was bi-directional: Including semantic associates in phonological lists or phonological associates in semantic lists resulted in parallel increases in false memories and the effect appeared to plateau after approximately three items were added. However, in a short-term task, these parallel effects were not observed: Inserting one or two semantic associates in a phonological list did not affect false memory rates, whereas inserting one or two phonological associates in a semantic list caused a dramatic increase in false memory rates (McBride et al., 2019). These studies show that replacing semantic associates in standard DRM lists with phonological associates not only increases false memory for the CI, but it also creates an over-additive effect on false memory. These findings highlight the separate contributions of semantic and phonological similarity to false memories.

Additional evidence for the role of structural overlap between list items and CIs comes from work using non-word stimuli (Zeelenberg et al., 2005). Following study of lists of similar non-words, the occurrence of false alarms to non-studied non-word CIs that shared phonemes with the list items suggested that participants were relying on surface features of the presented materials to drive a memory decision. Using lists of pseudohomophones (e.g., *dreem, bedd, awaik*), Cortese et al. (2008) found reliable false memories for semantically related items (i.e., *sleep*). The fact that pseudo-homophones, which result in reliable priming effects in word recognition tasks (Lukatela and Turvey, 1991), resulted in semantic activation of the CI underscores the importance of phonological information and its role in activating associative information. Taken together, the results using phonologically and orthographically related items, whether words or non-words, suggest that this type of similarity does negatively affect memory accuracy. Furthermore, the evidence suggests that these effects are stronger at shorter delays, whereas more persistent false memories are observed for semantically related items. To our knowledge, phonological lists have not been used in studies assessing forgetting rates at delays of more than a few minutes.

Although we have focused on verbal materials, it is worth noting that similar effects of perceptual or structural similarity have been observed with non-verbal materials such as categorized pictures (e.g., Seamon et al., 2000; Koutstaal et al., 2001), faces (Shimane et al., 2020), and novel shapes (Koutstaal et al., 2003). However, even with these materials, the false memory effects appear to be mediated by conceptual knowledge. For example, in Koutstaal et al.'s (2003) study, older adults, who typically have higher false memory rates than younger adults, only made more errors when novel shapes were given verbal labels. Similarly, Wang et al. (2018) concluded that false memories from pictures are due to the contributions of conceptual information. Using abstract images as stimuli, Sikora-Wachowicz et al. (2019) observed false memories in younger and older adults in a short-term memory task. Older adults in particular appeared to have increased difficulty discriminating studied items from foils, although this effect was observed for both targets and lure items, suggesting a more generalized deficit in sensitivity.

Given the parallels between the NAM (Luce and Pisoni, 1998) and network models such as Collins and Loftus' model [1975; see also Anderson (1983), Steyvers and Tenenbaum (2005)], which rely on spreading activation principles, at a general level, these findings are consistent with the AMF. Specifically, the basic assumption that neighbors in a network activate one another and result in increased familiarity or accessibility can accommodate both semantic and lexical networks. Although the underlying activation mechanism is similar in accounts of phonological and semantic false memory, in some cases, the mechanisms appear different. For example, Ballou and Sommers (2008) failed to find a correlation between phonological and semantic false alarm rates. Tse et al. (2011) provided evidence that the activation mechanisms involved in semantic and phonological false memories are distinct by examining discriminability measures and observing that CIs from semantically related lists were less discriminable than associates from the same list, whereas the opposite was true for phonologically related CIs. Furthermore, when a remember/know task (Tulving, 1985) designed to assess recollection and familiarity is employed, semantic lists yield more *remember* false alarms (e.g., Gallo et al., 1997; Geraci and McCabe, 2006), whereas phonological lists yield more *know* false alarms. This suggests that participants are basing their judgments on different information in the two cases, which might, in turn, reflect the fact that semantic lists drive attention to conceptual relations and thus more vivid and detailed memories, whereas phonological lists drive attention to surface features and less vivid memories (akin to a level-of-processing effect; Craik and Lockhart, 1972). In general, however, models based on activation processes can accommodate the results from phonologically related lists.

FTT, conversely, has more difficulty accommodating these results, given that gist traces are typically assumed to be derived from shared meaning. It is plausible that a structural similarity gist could be extracted; however, if that were the case, because gist is more persistent than verbatim traces, the reversal of false memory effects as a function of retention interval might be more difficult for this theory to accommodate. In fact, in recent work by one of the lead proponents of FTT, she claimed that “verbatim memory is memory for surface form, for example, memory representations of exact words, numbers and pictures. Gist memory is memory for *essential meaning* [emphasis added], the ‘substance' of information irrespective of exact words, numbers, or pictures” (Reyna, 2012, p. 333). Thus, lures that are similar to list items along dimensions that are distinct from the meaning of the items, should not be falsely remembered based on this definition of gist. An alternative to false memories based on gist might be through a process of trace disintegration, whereby verbatim traces decay and are recombined incorrectly during a retrieval process. However, using a modeling approach to estimate the contributions of gist and verbatim traces, Nieznański et al. (2019) concluded that the latter account is unlikely and that a more plausible account is that gist traces can emerge based on patterns of perceptual and surface similarity. However, even if one accepts that a “surface” or pattern gist is extracted, hyper-additive effects of phonological associate additions to DRM lists are also difficult to explain unless there is an assumption that two different gist traces are stronger than one.

More problematic for both AMF and FTT are non-word data. Because non-words, by definition, do not have a pre-existing memory trace and do not have meaning, it seems implausible that an activation model could readily accommodate them, given the relative dependence of this approach on pre-existing representations. GMMs, however, could readily account for these, as well as similar findings, by relying on trace similarity. Where GMMs have difficulty, though, is in explaining dissociations in false memories for phonological and semantic lists across short- and long-term delays. In GMMs, it is assumed that features of stored traces decay or are interfered with over time with no distinction made in the type of feature. However, other feature matching models, such a Nairne's (1990) feature model of short-term memory allow for weighting of different types of features over time. If such weighting is assumed, then GMMs can more readily account for the different results found at short- and long-term delays with different types of lists.

## Conclusions

In this review, we have considered the different ways that “similarity” has been defined with an emphasis on how these definitions affect the creation of false memories. This has shown how complex and multi-faceted a concept “similarity” is in this area of research. Generally speaking, memory is affected by all of the definitions considered here: semantic associations, feature overlap, phonological associations, and orthographic/lexical associations. Further, these effects are mostly age-invariant, as many studies have shown similar effects for young and older adults.

The results across a variety of paradigms and tests provide partial support for many theoretical explanations of false memory phenomena, but none of the theories readily account for all of the results. Specifically, when the word lists consisted of items that were indirectly related to the CI through a mediator but shared no direct relationship to the CI, false memories were most consistent with activation models, which assume that activation spreads through the network in a graded fashion. Because these lists do not have a common gist or many shared features, fuzzy-trace and global-matching models cannot as easily account for the results without additional assumptions. GMMs might be able to predict mediated false memories if the representations of the list items share sufficient features with the CI or with items more strongly associated to the CI. Conversely, when list items were both associatively related and shared primitive semantic features with the CI, false memories were greater than when lists were purely associatively related. Such a finding is inconsistent with activation-monitoring accounts, which prioritize associative strength as a mechanism, but consistent with fuzzy-trace and global-matching models, which emphasize similarity in terms of meaning, features, or gist. The AMF, to account for the feature boost effects, might incorporate multiple levels of activation as a core mechanism. Such an assumption was explicit in Collins and Loftus's (1975) original framework, in which semantic and lexical level information is coded. Thus, if activation spreads along pathways reflecting associative or lexical relations and along separate pathways reflecting semantic or conceptual relations, and this activation is additive in nature, the feature boost could be explained. Finally, false memories observed with lists of phonologically or orthographically related items can be accounted for by activation models, if the assumption is that the networks involved in supporting memory performance are organized in terms of structural similarity as well as semantic/associative similarity. Global-matching models can also account for such findings, provided perceptual/structural features are stored, but only if feature weight is assumed to vary in order to explain dissociations across short- and long-term delays. Finally, because fuzzy-trace models generally assume that gist is based on shared meaning, such an account cannot as readily account for these findings. By including a gist trace that is based on structural similarity it is plausible that FTT could account for these findings, although more specification would be needed. In addition, the assumption of FTT is that gist traces are more persistent than verbatim traces; however, the fact that phonological information appears to decay rapidly might be problematic, in that it would involve different forms of gist with potentially different parameters.

Clearly, the present review fails to provide unequivocal support for any of the major theoretical approaches discussed here. One potentially fruitful avenue for future work would be to critically examine the extent to which the distinction between the effects of structural, lexical, and semantic properties—which have been at the core of memory research for decades (e.g., Craik and Lockhart, 1972)—is as well-defined as it is typically assumed to be. For example, early conceptualizations of memory systems assumed that short-term stores primarily maintained structural properties, whereas long-term stores depended on semantic properties [see Greene (2016), for a review]. Evidence from a number of paradigms (e.g., orthographic distinctiveness effects) in addition to the phonological false memories discussed here suggest that structural information is retained in long-term memory and is involved in reconstructive processes. Furthermore, numerous effects point to the maintenance of semantic information in short-term memory (e.g., proactive interference effects, short-term false memories). Although there are clearly important distinctions between meaning-based and perceptually-based properties of stimuli, the evidence presented here does suggest that in many ways such properties exert similar effects on memory. Thus, further examining the extent to which such properties contribute to veridical and false memory and how they might interact with one another could help constrain and clarify the theories proposed here. At the moment, GMMs appear to be the most flexible in accommodating the results, given the way in which they are assumed to represent memory traces as arrays of features that are not necessarily semantic or perceptual in nature. However, such models might need to further specify the additive effects of associative and categorical effects (i.e., the feature boost) and the ways in which perceptual traces appear to have different decay rates than semantic traces.

Before closing, we acknowledge that the present review did not examine important work and converging evidence from neuroscientific and neuropsychological approaches. The question of how similarity and relatedness are represented has a long and rich history within the field, from early studies on category-specific deficits [see Lambon Ralph (2014), for a review] to work on semantic dementia, which reflects a selective loss of semantic memory in conjunction with relative preservation of episodic memory (e.g., Rogers et al., 2004; Lambon Ralph and Patterson, 2008) to more recent studies examining the role of modality-specific in semantic representations (Martin, 2007). Given that false memories driven by semantic and/or associative relations likely rely on pre-existing semantic representations, which are either activated or relied upon to extract gist traces, a more profound understanding of how these substrates contribute to episodic false memories is important. Charest et al. (2014) noted that, although there is a substantial degree of similarity across individuals in how objects are represented neutrally, idiosyncratic differences at the individual level could be predicted using imaging techniques, suggesting that the nature of similarity and relatedness might be even more complex. Whether false memories differ as a function of such individual differences might be a fruitful avenue for further work and could advance our understanding of the acquisition and malleability of shared and idiosyncratic representations of concepts. Additional work relevant to the present review suggests that the anterior temporal lobe is a central component in the maintenance and processing of conceptual and semantic information and that, interestingly, it is more strongly involved in associative processing than in categorical processing (Díez et al., 2017). Specifically, transcranial direct current stimulation of this region caused a significant decrease in associative false memories while leaving categorical false memories unaffected. Thus, future reviews and empirical work should integrate evidence from multiple protocols.

In sum, we conclude that similarity and relatedness are critical elements in how we encode, store, and retrieve information from memory and that examining errors and distortions provides insight into the functions of memory and of the knowledge base that supports memory performance.

## Author Contributions

All authors contributed to the writing and editing of the manuscript.

## Conflict of Interest

The authors declare that the research was conducted in the absence of any commercial or financial relationships that could be construed as a potential conflict of interest.

## Footnotes

^1^Recognition analyses are reported on corrected false recognition, calculated by subtracting false alarms to CIs related to non-studied lists from false alarms to CIs related to studied lists (lists were counterbalanced across conditions). Thus, the analyses correct for differential baseline false alarm rates.
